# Molecular Design Concept for Enhancement Charge Carrier Mobility in OFETs: A Review

**DOI:** 10.3390/ma16206645

**Published:** 2023-10-11

**Authors:** Yang Zhou, Keke Zhang, Zhaoyang Chen, Haichang Zhang

**Affiliations:** Key Laboratory of Rubber-Plastics of Ministry of Education, Shandong Province (QUST), School of Polymer Science & Engineering, Qingdao University of Science & Technology, 53-Zhengzhou Road, Qingdao 266042, China; 2022020042@mails.qust.edu.cn (Y.Z.); 19862464740@163.com (K.Z.); 4022020002@mails.qust.edu.cn (Z.C.)

**Keywords:** OFETs, semiconductor layers, charge carrier mobility, n-type

## Abstract

In the last two decades, organic field-effect transistors (OFETs) have garnered increasing attention from the scientific and industrial communities. The performance of OFETs can be evaluated based on three factors: the charge transport mobility (μ), threshold voltage (V_th_), and current on/off ratio (I_on/off_). To enhance μ, numerous studies have concentrated on optimizing charge transport within the semiconductor layer. These efforts include: (i) extending π-conjugation, enhancing molecular planarity, and optimizing donor–acceptor structures to improve charge transport within individual molecules; and (ii) promoting strong aggregation, achieving well-ordered structures, and reducing molecular distances to enhance charge transport between molecules. In order to obtain a high charge transport mobility, the charge injection from the electrodes into the semiconductor layer is also important. Since a suitable frontier molecular orbitals’ level could align with the work function of the electrodes, in turn forming an Ohmic contact at the interface. OFETs are classified into p-type (hole transport), n-type (electron transport), and ambipolar-type (both hole and electron transport) based on their charge transport characteristics. As of now, the majority of reported conjugated materials are of the p-type semiconductor category, with research on n-type or ambipolar conjugated materials lagging significantly behind. This review introduces the molecular design concept for enhancing charge carrier mobility, addressing both within the semiconductor layer and charge injection aspects. Additionally, the process of designing or converting the semiconductor type is summarized. Lastly, this review discusses potential trends in evolution and challenges and provides an outlook; the ultimate objective is to outline a theoretical framework for designing high-performance organic semiconductors that can advance the development of OFET applications.

## 1. Introduction

Field-Effect Transistors (FETs) are active devices that utilize an electric field to regulate the conductivity of solid materials [1,2,3]. FETs play a crucial role in the microelectronics industry and facilitate the precise control of current within circuits. Nevertheless, the high cost has constrained the practical application and development of inorganic field-effect transistors [4,5,6,7,8,9]. In response to this challenge, scientists have introduced organic field-effect transistors (OFETs) as replacements for inorganic counterparts [10,11]. In contrast to inorganic field-effect transistors, organic field-effect transistors offer numerous advantages, including cost-effectiveness, exceptional flexibility, and the ability to be processed at low temperatures [12,13,14]. Thus far, high-performance organic semiconductors have been seamlessly integrated with other technologies, such as phototransistors, sensors, flexible displays, or energy-harvesting devices, to create multifunctional systems [15,16,17,18,19]. This also provides challenges and opportunities for creating multifunctional systems with organic semiconductors.

Generally, OFETs can be categorized into three types: p-type (hole transport), n-type (electron transport), and ambipolar (both hole and electron transport) [20]. The classification of OFETs is primarily based on the properties of the organic semiconductor layer [21,22,23,24,25,26]. The development of n-type and ambipolar OFETs is lagging behind that of p-type OFETs. This is mainly due to the fact that the radical anions of organic materials are more sensitive to water and oxygen in the air. In order to improve the stability and reliability of n-type and ambipolar OFETs, the devices were often protected by the internal gases such as nitrogen or argon gas [27,28]. In addition, the optimal energy levels also affect the stability of the n-type device whose LUMO energy level is usually higher than −4.6 eV. The n-type materials can be used not only for the preparation of OFETs but also for a wide range of other applications such as the electronic transport layer in the perovskite solar cells and organic light-emitting devices [29,30].Consequently, the key to developing high-performance OFETs lies in the design of a suitable organic semiconductor layer [31,32]. The primary determinants for enhancing the performance of organic semiconductors are the charge transport mobility (μ), threshold voltage (V_th_), and current on/off ratio (I_on/off_). In these factors, charge transport is the pivotal element driving the performance of OFETs [33,34,35,36,37]. Therefore, the selection of appropriate molecular design strategies is critical for enhancing the properties of the organic semiconductor layer and manufacturing high-performance OFETs.

In recent years, three prominent strategies have emerged for enhancing the charge mobility of organic semiconductor molecules: (i) extending π-conjugation, enhancing molecular planarity, and optimizing donor–acceptor structures to improve charge transport within the individual molecules [38,39,40,41]; and (ii) promoting strong aggregation, achieving well-ordered structures, and reducing molecular distances to enhance the charge transport between the neighboring molecules [42]. Moreover, in order to obtain a high charge transport mobility, the charge injection from the electrodes into the semiconductor layer is also important [43,44,45,46]. This alignment occurs because the suitable energy levels of frontier molecular orbitals match well with the work function of the electrodes, thereby forming an Ohmic contact at the interface.

In the development of OFETs, although there are many strategies for improving carrier mobility, in fact, there is still a lack of an effective set of guiding rules to assist researchers. Hence, in this review, it introduces a conceptual framework aimed at equipping researchers with the knowledge to enhance the efficiency of OFET device preparation. Herein, we introduce various molecular design concepts aimed at enhancing the charge carrier mobility, including both within the semiconductor layer and during the charge injection. In addition, we provide a summary of how to design or convert the type of semiconductor. Lastly, we discuss potential trends in evolution and challenges and provide an outlook. The ultimate goal is to create a theoretical framework for designing high-performance organic semiconductors while advancing the development of OFETs applications.

## 2. Backbone

Enhancing charge mobility needs an appropriate molecular structure as a prerequisite. Charge transport in the semiconductor layer is divided into inter- and intra-charge transport. Regarding the intra-charge carrier’s mobility, the charge mainly transports through the effective conjugate system such as the molecular backbone [47,48]. Therefore, the introduction of electron-withdrawing and -rich groups into molecules can enhance the semiconductor performance to some extent, since this method could improve the electron cloud distribution. In addition, expanding the π-conjugated system can significantly enhance the effective conjugation system, thus improving the charge transport mobility [49,50].

**Figure 1:**
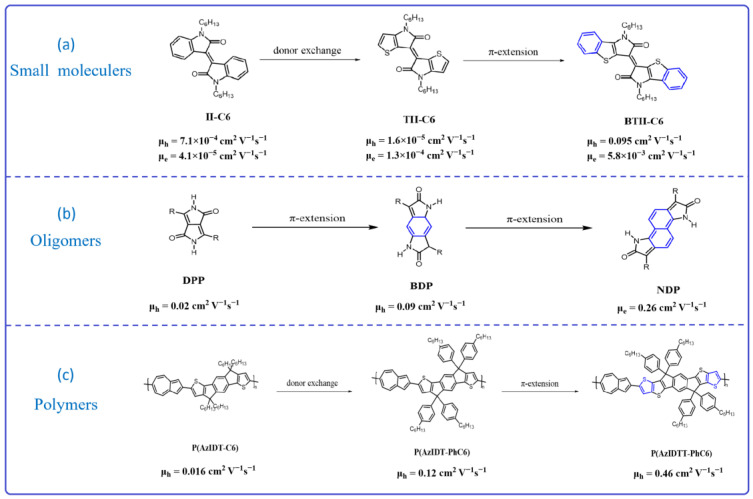
Chemical structures of the π-conjugation extension molecules based on small molecules, oligomers, and polymers as well as their charge transport mobility.

Tsukasa Hasegawa and his colleagues [51] synthesized three molecules based on isoindigo, which are named isoindigo (II-C6), thieoisoindigo (TII-C6), and benzothienoisoindigo (BTII-C6) (Figure 1a). Compared to II-C6, TII-C6 used the thiophen group instead of the benzene ring, which resulted in the TII-C6 having a strong electron-rich ability. In addition, the BTII-C6 exhibits a π-conjugation extension behavior compared to the other small molecules. As a consequence, the BTII-C6 presents a significantly enhanced charge transport mobility—up to a μ_h_ value of 0.095 cm^2^ V^−1^ s^−1^ and a μ_e_ value of 5.8 × 10^−3^ cm^2^ V^−1^ s^−1^—which is much higher than that of II-C6 (μ_h_ = 7.1 × 10^−4^ cm^2^ V^−1^ s^−1^ and μ_e_ = 4.1 × 10^−5^ cm^2^ V^−1^ s^−1^, and TII-C6 (μ_h_ = 1.6 × 10^−5^ cm^2^ V^−1^ s^−1^ and μ_e_ = 1.3 × 10^−4^ cm^2^ V^−1^ s^−1^). Recently, Deng et al. [52] synthesized three π-conjugated oligomers based on diketopyrrolopyrrole (DPP), benzodipyrrolidone (BDP), and naphthodipyrrolidone (NDP) (Figure 1b). There are additional benzene or naphthalene rings in the core for BDP or NDP compared to isoDPP, which will result in a π-extension system and increase the intra-charge transport within the single molecules. The results show that, with the π-conjugation extension system, the highest occupied molecular orbital (HOMO) energy levels were increased and the LUMO energy levels were decreased, which can result an Ohmic contact. The authors used these three oligomers to build crystal OFETs. DPP and BDP-based oligomers exhibit p-type behavior, with a hole mobility of 0.02 cm^2^ V^−1^ s^−1^ and 0.09 cm^2^ V^−1^ s^−1^, while oligomers based on NDP show n-type behavior, with an electron mobility of up to 0.26 cm^2^ V^−1^ s^−1^. This work demonstrated that an extension π-conjugation system is not only a useful strategy for increasing the performance of semiconductors but could also alter its charge transport type. Regarding polymers, the effective conjugation system is large due to its long polymer backbone. Extending the π-conjugation of the repeating units is also beneficial for enhancing semiconductor properties. Hou and his colleagues [53] synthesized three conjugated copolymers between the 2,6-azulene unit and electron indacenodithiophene (IDT) units, which are named P(AzIDT-C6), P(AzIDT-PhC6), and P(AzIDTT-PhC6) (Figure 1c), respectively. Compared with P(AzIDT-C6) and P(AzIDT-PhC6), P(AzIDTT-PhC6) features an extension of π-conjugation with two additional thiophene-fused rings. The results indicate that P(AzIDTT-PhC6) exhibits superior performance, with a hole mobility reaching 0.46 cm^2^ V^−1^ s^−1^, whereas P(AzIDT-C6) and P(AzIDT-PhC6) exhibit hole mobilities of only 0.016 cm^2^ V^−1^ s^−1^ and 0.12 cm^2^ V^−1^ s^−1^, respectively. The expanded conjugated system promotes charge transfer within the single molecule, resulting in a significant enhancement of carrier mobility. Based on the above observation as well as the published literature [54,55], π-conjugation extension in the material’s backbone (including small molecules, oligomers, and polymers) is a simple and useful strategy for designing high-performance semiconductors.

**Figure 2:**
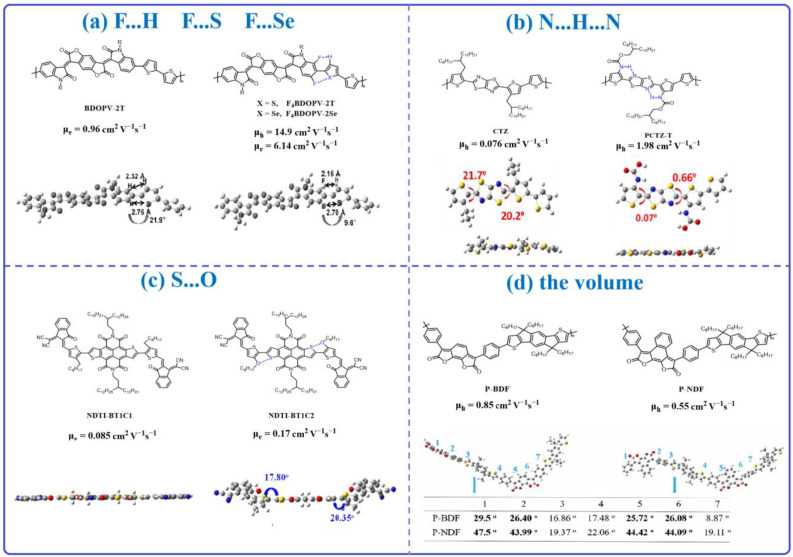
Chemical structures of the molecules with non-covalent bonds interaction to adjust their backbone’s planarity.

Besides extending the π-conjugated length, modifying the backbone’s planarity is a simple and useful design strategy in OFETs. This strategy could not only enhance the efficiency of the conjugation length but also improve the order and regularity of molecular arrangement and promote π-π stacking, which results in enhancing charge carrier mobility [56,57,58]. Based on these theoretical foundations, in the past few years, scientists have made many attempts to introduce the non-covalent bond into the material’s backbone to adjust the material’s backbone’s planarity, such as F…S, F…H, N…H…N, S…O, and so on. Zheng et al. [59] synthesized three polymers, BDOPV-2T, F4BDOPV-2T, and F4BDOPV-2Se. Compared with BDOPV-2T, there is a fluorine atom existing in the BDOPV units for F4BDOPV-2T and F4BDOPV-2Se, which results in the F…H and F…S/F…Se interaction for F4BDOPV-2T and F4BDOPV-2Se, respectively. The non-covalent bonding further adjusts the polymer backbone’s planarity, which optimizes the torsion angle between the acceptor part (BDOPV) and flanked thiophene, decreasing it from 21.9° (BDOPV-2T) to 9.6° (F4BDOPV-2T) (Figure 2a). As a consequence, the F4BDOPV-2T and F4BDOPV-2Se present a significantly improved electron transport mobility up to 14.9 cm^2^ V^−1^ s^−1^ and 6.14 cm^2^ V^−1^ s^−1^, which is almost ten times that of BDOPV-2T (μ_e_ = 0.96 cm ^2^ V^−1^ s^−1^). In addition, the stability of the device is also improved. After 30 days, the electron mobility of the F4BDOPV-2T device is only 40% lower than the original mobility, while the μ_e_ of BDOPV-2T is almost 75% lost. The improved stability is ascribed to the strong interchain interaction of the F…S, F…H, and F…Se results, the more planar backbone, the ordered molecular packing, as well as the small π-π stacking distance, which increase the oxygen penetration barrier. Recently, Liu et al. [60] designed two polymers, CTZ and PCTZ-T. Both polymers present the same backbone, but there is a nitrogen atom in the alky chain of the PCTZ-T. The existing nitrogen atom could form an N…H…N RAHB interaction, which locks the thiophene units with the thiazolothiazole units. As a consequence, the torsion angle between the thiophene units and thiazolothiazole groups is significantly decreased from 20.2° (CTZ) to 0.07° (PCTZ-T) (Figure 2b). The author fabricated the OFET device using these two polymers as the semiconductor layer. The result indicates that the μ_h_ of CTZ is only 0.076 cm ^2^ V^−1^ s^−1^, while the value of PCTZ-T is almost 30 times increased, which is up to 1.98 cm ^2^ V^−1^ s^−1^. Ran et al. [61] introduced the S…O interaction into the polymer backbone. The results indicate that the torsion angle is decreased from 20.35° to almost 0° due to the S…O interaction (Figure 2c). Eventually, the electron mobility of NDTI-BT1C2 reaches 0.17 cm^2^ V^−1^ s^−1^, and the sulfur–oxygen interactions form a more regular plane, whereas the electron mobility of NDTI-BT1C1 is only 0.085 cm^2^ V^−1^ s^−1^. Beside the above-mentioned interaction, some other non-convent bonds are also widely studied, such as N…S, B←N [62,63], and so on.

In most cases, researchers adjust the material’s backbone’s planarity through the non-convent bonds but ignore the volume of the groups. Recently, Zhang’s group [64] studied the benzo/naphthodifuranone-based polymers P-BDF and P-NDF. Both polymers exhibit a similar backbone, except there is an additional benzene ring existing in the P-NDF, which results in P-BDF having a more planar backbone conformation (Figure 2d) due to its smaller conjugated core size, and P-NDF features a perpendicular-extended main chain structure. Due to the perpendicular π-conjugation extension of the P-NDF, it should present high charge transport mobility. But the result found that the μ_h_ of P-NDF is only 0.55 cm^2^ V^−1^ s^−1^, while that of P-BDF is up to 0.85 cm^2^ V^−1^ s^−1^. This high value of P-BDF is ascribed to the planar backbone resulting in stronger aggregation as well as the short π-π distance. This work indicates that the main chain coplanarity of polymer semiconductors is more essential than the sole extension of π-conjugations (especially at the perpendicular direction of polymer main chains) for the design of high-performance OFET materials. The decrease in the volume of the donor or acceptor groups is a simple and useful strategy for designing high-performance semiconductor materials, such as thiophene units, instead of the benzene ring [65].

**Figure 3:**
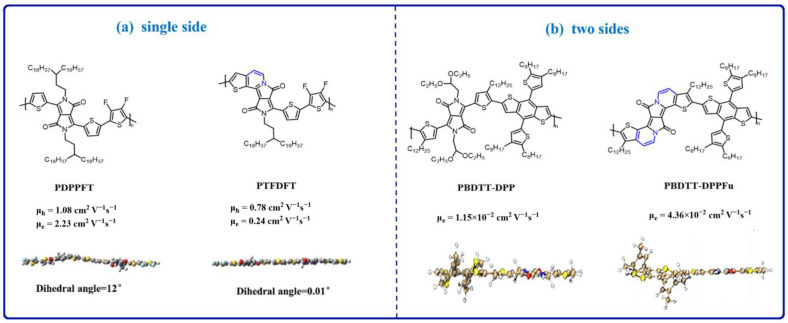
Chemical structures of the molecules with ring fusing to adjust their backbone’s planarity.

Introducing a non-covalent bond and reducing the value of the units can significantly reduce the twisting angle and improve the material’s backbone planarity, but it is difficult to reach 0°. Very recently, Shi et al. [66] found that totally planar structures can be obtained through the ring fusion method. The author studied two polymers, PDPPFT and PTFDFT. Compared to PDPPFT, PTFDFT has high conjugation and planarity by fusing the alkoxy chain situated on a single side of the DPP with adjacent thiophene groups, which results in the twist angle between the DPP core and the thiophene units improving from 12° to 0.01° (Figure 3a). Compared to PDPPFT, PTFDFT has a more uniform molecular plane and a higher degree of conjugation, resulting in enhanced performance. It demonstrates ambipolar OFET behavior with a μ_h_ value of 1.08 cm^2^ V^−1^ s^−1^ and a μ_e_ value of 2.23 cm^2^ V^−1^ s^−1^, while PDPPFT exhibits a μ_h_ value of 0.78 cm^2^ V^−1^ s^−1^ and a μ_e_ value of 0.24 cm^2^ V^−1^ s^−1^. After the ring closing, the charge transport mobility is significantly increased, typically for the μ_e_. It should be noticed that after the ring closing, the solubility of the polymers is decreased due to the reduction of the alky side chain. Later, Zhuang et al. [67] studied the two sides’ ring fusion regarding the DPP that obtained two polymers, PBDTT-DPPFu and PBDTT-DPP. Compared to PBDTT-DPP, PBDTT-DPPFu has high conjugation and planarity (Figure 3b). Both polymers present n-type behavior, with 1.15 × 10^−2^ cm^2^ V^−1^ s^−1^ for PBDTT-DPPFu and 4.36 × 10^−3^ cm^2^ V^−1^ s^−1^ for PBDTT-DPP. After the ring closing, the electron transport mobility increased by almost three times. These observations reveal that totally planar structures can be designed through the ring fusion method.

Consequently, the extension of the π-conjugation system and enhancing the planarity of the backbone are simple and useful strategies for building high-performance semiconductor materials with high charge transport mobility. Reducing the volume of the units and ring fusion are effective methods for adjusting the molecular backbone’s planarity. Undoubtedly, these methods have proven effective in the context of current developments, and their potential for significant applications is anticipated in the future advancement of OFETs technology.

## 3. Side Chains Engineering

**Figure 4:**
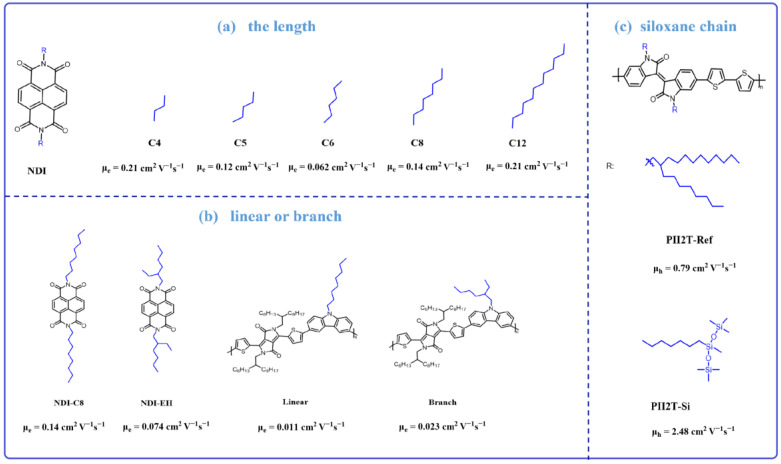
Chemical structure based on the molecules with different-type alky-chains as well as their charge transport mobility.

It is widely recognized that conjugated polymers containing aromatic rings exhibit greater rigidity and reduced pliability, resulting in poor solubility. In contrast, alkyl chains offer enhanced pliability, and their judicious incorporation into polymers can significantly improve solubility [68,69]. In general, the inclusion of long alkyl chains enhances solubility; however, it tends to diminish intermolecular stacking, potentially leading to reduced carrier mobility. Conversely, short alkyl chains promote closer intermolecular stacking but cannot provide the same level of solubility as long alkyl chains. Welford et al. [70] synthesized a series of molecules with different lengths and shapes of side chains-based naphthalene diimide (NDI), which included NDI-C4, NDI-C5, NDI-C6, NDI-EH, NDI-C8, and NDI-C12 (Figure 4a). Among these molecules, NDI-C4, NDI-C5, NDI-C6, NDI-C8, and NDI-C12 contain linear side chains with four (C4), five (C5), six (C6), eight (C8), and twelve (C12) carbon atoms, respectively, while NDI-EH has eight (C8) branched side chains. Comparing these linear side chains, both the shortest chain molecule NDI-C4 (μ_e_ = 0.21 cm^2^ V^−1^ s^−1^) and the longest chain molecule NDI-C12 (μ_e_ = 0.21 cm^2^ V^−1^ s^−1^) showed the best performance, while the low mobilities semiconductor materials are observed among the intermediate chain lengths (NDI-C5: μ_e_ = 0.12 cm^2^ V^−1^ s^−1^, NDI-C6: μ_e_ = 0.062 cm^2^ V^−1^ s^−1^, NDI-C8: μ_e_ = 0.14 cm^2^ V^−1^ s^−1^). The mobility trends in terms of the competing factors of film uniformity and crystal packing were interpreted. Short side chains promote a more favorable lateral stacking of NDI units within the unit cell, while long side chains promote a more uniform thin-film morphology. This comparison inspires us to refrain from assessing the optimal efficiency based solely on the length of the alkyl chain in the molecular backbone. Instead, it urges us to strike a balance among solubility, intermolecular π-π stacking, and crystallization properties simultaneously. Comparing NDI-C8 and NDI-EH (Figure 4b), their alkyl chains are both composed of eight carbon atoms, but they are distinguished by the fact that NDI-C8 has a linear side chain, while NDI-EH has a branched side chain. Notably, NDI-C8 obtains an electron mobility of 0.14 cm^2^ V^−1^ s^−1^, which is almost two times higher than that of NDI-EH (μ_e_ = 0.074 cm^2^ V^−1^ s^−1^). It is revealed that, with the same number of alkyl chain carbons, the linear side chain can form better intermolecular π-conjugation than the branched side chain.

Although the majority of molecules adhere to this trend, there exist exceptional cases. Zhang and colleagues [71] synthesized two molecules based on DPP and carbazole, which are named linear and branch (Figure 4b), respectively. These two polymers present almost the same polymer backbone and chemical structures, except the side alkyl chains are linear or branch. The XRD as well as the optical absorption studies indicate that the linear polymer exhibits stronger aggregation than the branch one. Normally, strong aggregation is beneficial for charge transport between individual molecules. Thus, the linear polymer having a high charge transport mobility is expected. However, the linear polymer exhibits a hole mobility of 1.1 × 10^−2^ cm^2^ V^−1^ s^−1^, which is lower than the 2.3 × 10^−2^ cm^2^ V^−1^ s^−1^ observed for the branch polymer. The GPC measurement shows that the branch polymer has a high molecular weight, which might be due to the fact that the branch polymer exhibits improved solubility compared to linear ones. This is the reason why the branch polymer’s performed is better than that of the linear polymer. We should consider both molecular packing and molecular weight when we consider using linear or branch alky chains.

Besides enhancing the solubility, alky-chain engineering could also endow the martials with some other functional ability. For instance, alkyl chains incorporating silica can offer exceptional flexibility and further optimize intermolecular stacking. Mei and colleagues [72] synthesized an alkyl chain terminating in siloxane groups and affixed it to the isoindigo molecular system (Figure 4c). The introduction of this ultra-flexible molecular chain had a reduced impact on the stacking of the rigid backbone, resulting in a π-π stacking distance of 3.58 Å, while the reference polymer showed a distance of 3.76 Å. PII2T-Si displayed an impressive hole mobility of 2.48 cm^2^ V^−1^ s^−1^, significantly surpassing the modest 0.79 cm^2^ V^−1^ s^−1^ exhibited by a conventional all-carbon chain. This work reveals that introducing special atoms, such as phosphonate [73], -S, and -N [74], into the alkychian could improve the materials packing in the solid state, resulting in a high inter-charge transport mobility.

**Figure 5:**
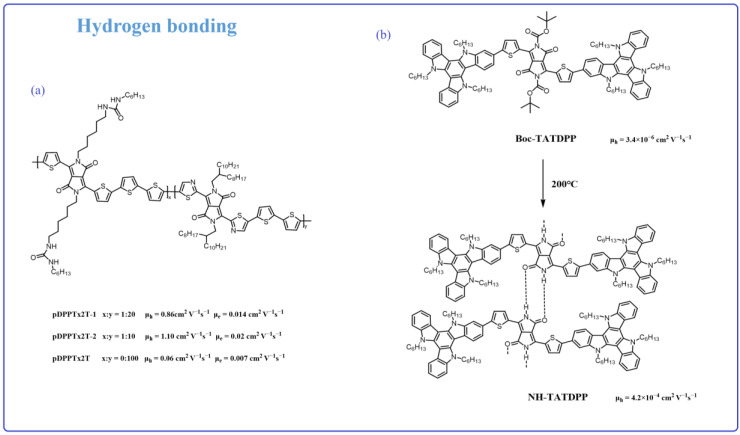
Chemical structure based on the molecules with hydrogen bonding association as well as their charge transport mobility.

Recently, numerous studies have verified that alky chain engineering incorporating hydrogen bonding interactions is a beneficial strategy, which can result in materials with self-assemble ability. This improves the molecular packing and shortens the π-π distance, resulting in a high charge transport mobility [75,76,77,78,79,80,81]. Ma et al. [82] synthesized three new thiazole-flanked DPP-based polymers, pDPPTz2T, pDPPTz2T-1, and pDPPTz2T-2 (Figure 5a), with urea-containing linear side chains. The hydrogen bonding interaction (NH…OC) could be formed through the urea units. The ratios of urea-containing linear side chains and full carbon chain moieties in pDPPTz2T, pDPPTz2T-1, and pDPPTz2T-2 were 0:100, 1:20, and 1:10, respectively, which means that there is hydron bonding interaction existing in the pDPPTz2T-1 and pDPPTz2T-2, but not in pDPPTz2T. The AFM image shows that intermolecular hydrogen bonding induced better accumulation, which leads to the formation of larger aggregation for pDPPTz2T-2. With the proportion of urea groups increasing, the hole mobility and electron mobility of the corresponding polymers are enhanced to some extent (pDPPTz2T:μ_h_ = 0.06 cm^2^ V^−1^ s^−1^, μ_e_ = 7 × 10^−3^ cm^2^ V^−1^ s^−1^; pDPPTz2T-1:μ_h_ = 0.86 cm^2^ V^−1^ s^−1^, μ_e_ = 0.014 cm^2^ V^−1^ s^−1^; pDPPTz2T:μ_h_ = 1.10 cm^2^ V^−1^ s^−1^, μ_e_ = 0.02 cm^2^ V^−1^ s^−1^). This work demonstrates the hydrogen bonding effects on the alky chain. However, if the hydrogen bonding directly worked on the polymer backbone, it will significantly affect the conjugation system as well as the electron cloud distribution.

The alky chain could increase the material’s solubility and enable its solution processing but will result in materials packing disorder. In addition, it cannot enhance the materials’ backbone conjugation systems. In the past few years, researchers found that the Boc unit is broken down during the thermal annealing process. Thus, Boc groups could endow the conjugated materials with a good solubility in most common organic solvents, resulting in a solution processing ability. In most reported articles, the Boc unit is substituted on the amide units (NH). Once the OFET device is prepared, through the thermal annealing processing, the Boc units could be removed; meanwhile, the device based on the materials without any alky-chain is obtained. In addition, the presence of amide units can form hydrogen bonds with the carbonyl groups of neighboring molecules (NH…OC), resulting in materials with self-assembly capabilities. Mula et al. [83] successfully synthesized Boc-TATDPP and NH-TATDPP (Figure 5b). NH-TATDPP was obtained by annealing Boc-TATDPP at 200 °C. Once the Boc group is removed, the exposed hydrogen bonds have the potential to engage in intermolecular interactions with the carbonyl group. The hydrogen bonding between NH-TATDPP helped regarding an ordered self-assembly, with the intermolecular π-π stacking distance reduced from 1.63 nm to 0.3 nm (Boc-TATDPP). Compared to Boc-TATDPP, NH-TATDPP shows better crystallization properties. The peak of the casted film was from 2θ = 5.413° (Boc-TATDPP) to 2θ = 29.55° (NH-TATDPP) (XRD sepctra). In addition, the hydrogen bonding association results in the oligomer forming a liner shape-liked polymer, which also affects its conjugation system. In the end, the hole mobility of hydrogen-bonded NH-TATDPP (μ_h_ = 4.2 × 10^−4^ cm^2^ V^−1^ s^−1^) was enhanced by two orders of magnitude compared to that of Boc-TATDPP (μ_h_ = 3.4 × 10^−6^ cm^2^ V^−1^ s^−1^). Besides small molecules, oligomers as well as the polymers containing Boc groups also present the same properties with a self-assembling ability, enhancing the charge transport mobility [84,85,86]. Consequently, these studies demonstrate that introducing hydrogen bonding side chains is a meaningful strategy for enhancing charge mobility. Furthermore, we can rationalize the incorporation of hydrogen bonds at various locations within the molecule using distinct approaches.

In summary, alky chain engineering plays a crucial role in the molecular design concept for high-performance semiconductor materials. Achieving precise control over the alkyl chain’s length and shape (linear or branched), the incorporation of specific groups (-Si, -S, -N), or the implementation of a hydrogen bonding system in side-chain engineering offers opportunities to enhance molecular stacking and boost carrier mobility. This also enables the harmonization of semiconductor layer solubility with high efficiency, providing a crucial foundation for the fabrication of efficient OFET devices.

## 4. Ohmic Contact

The essential components of an OFET device include the semiconductor layer and the electrodes. The organic semiconductor layer is directly in contact with the electrodes, allowing for the transmission of charge between the organic semiconductor layer and the electrodes [87,88,89]. Therefore, in the process of enhancing charge mobility within the organic semiconductor layer, it is essential to design materials with suitable frontier molecular orbital levels (FMOs) that align with the work function of the electrodes, ultimately forming an Ohmic contact at the interface [90,91]. Typically, when the HOMO of a molecule aligns with the Fermi energy levels of the electrodes, it can establish efficient hole-transport pathways and facilitate the creation of efficient p-type OFET devices. Accordingly, when the LUMO of a molecule aligns with the Fermi energy levels of the electrodes, it can form efficient electron-transport channels, and it is a critical factor in advancing the development of n-type OFET devices. In the operation of an OFET device, charges are injected from one electrode, traverse the semiconductor layer, and return to the opposite electrode, completing a cycle. Several losses occur in this charge transport process, with the most critical ones being charge injection at the electrode–semiconductor interface and charge migration within the semiconductor layer [92]. While recent research has concentrated on optimizing semiconductor efficiency, the significance of establishing ohmic contact between the electrodes and the semiconductor layer has been frequently overlooked, which often plays a pivotal role in OFET device performance.

**Figure 6:**
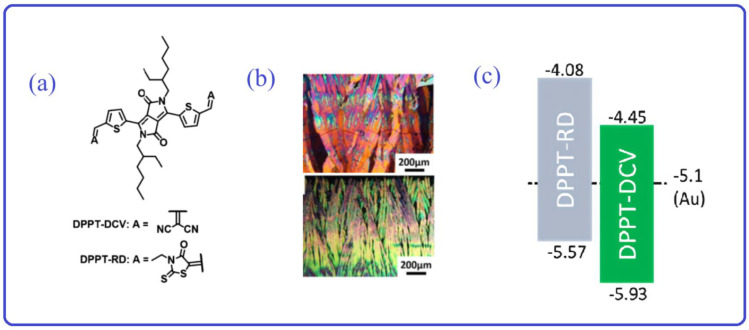
(**a**) The chemical structure of DPPT-DCV and DPPT-RD. (**b**) POM images of DPPT-RD and DPPT-DCV crystal arrays on Si wafers. Adapted with permission from [98]. (**c**) The HOMO/LUMO energy levels of DPPT-DCV and DPPT-RD. Adapted with permission from [98].

To improve charge carrier injection properties in the organic semiconductor layers, especially in n-type OFETs, the organic semiconductor layers should have suitable HOMO and LUMO. A low LUMO energy level can produce an excellent match with the Fermi energy level of the metal electrode, thus reducing the barrier for electron injection. In general, the introduction of fluorine, cyano, and some other electron-deficient groups into the molecular system can enhance the electron-deficient properties of the materials [93,94,95,96,97], thus obtaining a lower LUMO energy level. This results in a good match with the metal electrode, which optimizes the interface between the semiconductor layer and the electrode. San-Lien Wu and his coworkers developed two DPP-based conjugated molecules, DPPT-RD and DPPTDCV, which contain 3-ethyl rhodanine (RD) and dicyano-2-vinyl (DCV) (Figure 6a) [98]. Compared with DPPT-DCV, DPPT-RD significantly improves the crystallization of the film because of the RD substituent group (Figure 6b). Notably, the HOMO and LUMO of DPPT-RD have been significantly enhanced (Figure 6c), rendering them more compatible with the Fermi energy levels of the electrodes, which are suitable for holes (2.16 × 10^−2^ cm^2^ V^−1^ s^−1^) and electron (7.27 × 10^−2^ cm^2^ V^−1^ s^−1^) injection from the Au source-drain electrodes. However, due to the poor alignment Fermi energy levels between the DPPT-DCV and the metal electrode, carriers encounter significant ohmic resistance during migration. As a result, DPPT-DCV exhibits a weak p-type characteristic with an electron mobility of 1.84 × 10^−2^ cm^2^ V^−1^ s^−1^. Therefore, from the semiconductor layer’s perspective, the design of molecules closely aligned with the Fermi energy level of the metal electrodes can effectively mitigate ohmic resistance during carrier transfer, resulting in enhanced OFET performance.

**Figure 7:**
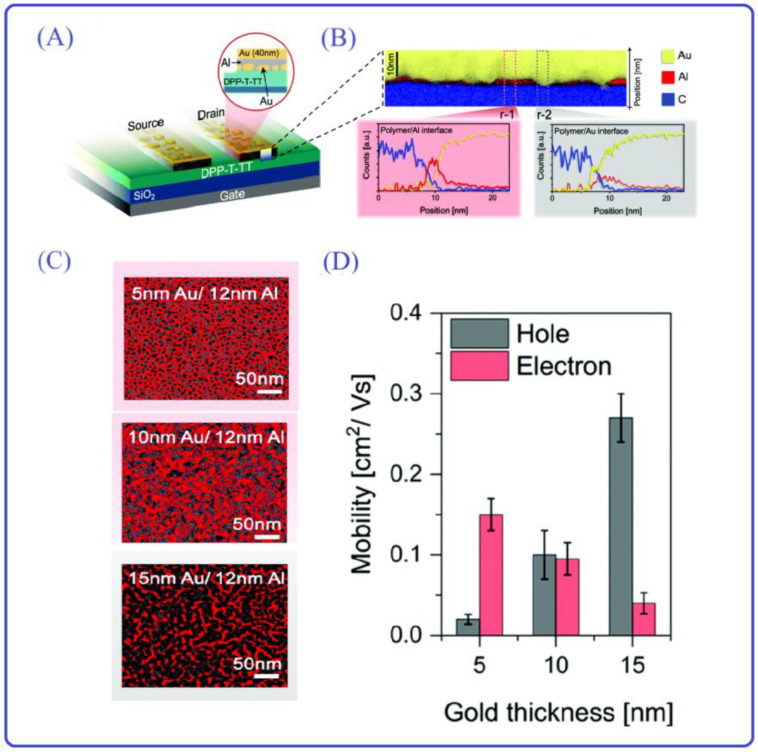
(**A**) Schematic illustration of the top-contact bottom-gate OFET device. Adapted with permission from [102]. (**B**) STEM-EDX cross-section image (**top**) and elemental maps (**bottom**) of the OFET device with the metal–mosaic electrodes. Adapted with permission from [102]. (**C**) Surface morphology of Au/Al electrodes with different ratios in OFET. Adapted with permission from [102]. (**D**) The corresponding electron and hole mobilities extracted from the n- and p-type devices, respectively. Adapted with permission from [102].

In addition, for the purpose of the formation of the Ohmic contact, it is possible not only to ensure a rational alignment of semiconductor molecules with metal electrodes but also to consider the design of diverse electrodes from the metal electrodes’ perspective. This approach aims to achieve superior ohmic contact with the semiconductor layer’s molecules. In the charging process of holes and electrons, organic semiconductors usually support ambipolar transport. However, the charge injection is symmetric due to the inherent misalignment of the electrode work function with both conducting levels of the organic semiconductors [99,100,101]. Starting from the perspective of electrodes, Sarkar et al. reported a new electron design strategy for constructing ambipolar OFETs. The electrode is composed of Al and Au, which form a mosaic-like structure composed of islands of two metals with high and low work functions (Figure 7A,B) [102]. With the suitably applied bias, the Au domain allows for the hole injecting to the HOMO. Respectively, the electron can inject into the LUMO. Thus, this electron can be simultaneously to the source and drain, which have high on/off ratios. Furthermore, alterations in the Al-to-Au ratio (Figure 7C) result in changes in the n/p-type behavior. For instance, (i) Al (12 nm)/Au (5 nm) exhibits pronounced n-type characteristics; (ii) Al (10 nm)/Au (12 nm) yields a well-balanced combination of n- and p-type performance; and (iii) Al (10 nm)/Au (12 nm) demonstrates marked p-type behavior (Figure 7D). This occurs primarily because the ratios of various metals can be adjusted to align with the Fermi energy levels of the electrodes. This leads to varying degrees of alignment with the HOMO and LUMO energy levels of the semiconductor layer, consequently affecting the formation of ohmic contacts for holes and electrons. The new electrode design concept provides a great solution for electronic devices that require ambipolar OFETs, such as light-emitting transistors, organic memory devices, and so on.

While many organic semiconductor layer materials currently exhibit high efficiencies, converting them to high-efficiency OFET devices has significant challenges, primarily in dealing with the ohmic contact problem. Consequently, both from the electrode and semiconductor layer perspectives, there is a pressing need to enhance passivation, minimize ohmic resistance, and mitigate unnecessary carrier losses. This is the primary goal for the efficient preparation of OFET in the future.

The performance of the OFETs, as summarized in this review, are provided in Table 1.

## 5. Conclusions and Outlook

OFETs address the shortcomings of inorganic field-effect transistors, including their high cost, environmental impact, and lack of flexibility. Furthermore, the advancement of OFET technology has enabled the creation of flexible electronic devices. In recent years, OFETs have played pivotal roles in our daily lives and have become essential in various fields, including flexible displays, electron-skin technology, and numerous advanced applications. As these fields continue to evolve, the demand for high-performance OFETs with a high charge transport mobility has grown significantly.

Despite the long development history of OFETs, several challenges continue to impede their practical application. These challenges include lower charge mobility when compared to that of inorganic field-effect transistors and issues of stability, particularly in the case of n-type and ambipolar OFETs. In addition, environmentally friendly and sustainable materials and manufacturing processes for organic semiconductors are also challenging. Given these challenges, this review focuses primarily on the molecular design concepts for achieving high-performance n-type and ambipolar OFETs. This encompasses considerations related to the backbone, side chains, and electrodes. The ultimate objective is to provide a theoretical framework for designing high-performance n-type and ambipolar OFETs, thereby advancing their development for practical applications.

## Figures and Tables

**Table 1:** The important parameters of the OFETs.

OFETs	μ_(h)max(cm_^2^_V_^−1^_s_^−1^_)_	μ_(e)max(cm_^2^_V_^−1^_s_^−1^_)_	Ref.
II-C6	7.1 × 10^−4^	4.1 × 10^−5^	[51]
TII-C6	1.6 × 10^−5^	1.3 × 10^−4^	[51]
BTII-C6	0.095	5.8 × 10^−3^	[51]
DPP	0.02		[52]
BDP	0.09		[52]
NDP		0.26	[52]
P(AzIDT-C6)	0.016		[53]
P(AzIDT-PhC6)	0.12		[53]
P(AzIDTT-PhC6)	0.46		[53]
BDOPV-2T		0.96	[59]
F4BDOPV-2T		14.9	[59]
F4BDOPV-2Se		6.14	[59]
CTZ	0.076		[60]
PCTZ-T	1.98		[60]
NDTI-BT1C1		0.085	[61]
NDTI-BT1C2		0.17	[61]
P-BDF	0.85		[64]
P-NDF	0.55		[64]
PDPPFT	0.78	0.24	[66]
PTFDFT	1.08	2.23	[66]
PBDTT-DPP		4.36 × 10^−3^	[67]
PBDTT-DPPFu		1.15 × 10^−2^	[67]
NDI-C4		0.21	[70]
NDI-C5		0.12	[70]
NDI-C6		0.062	[70]
NDI-EH		0.074	[70]
NDI-C8		0.14	[70]
NDI-C12		0.21	[70]
Linear	1.1 × 10^−2^		[71]
Branch	2.3 × 10^−2^		[71]
PII2T-Ref	0.79		[72]
PII2T-Si	2.48		[72]
pDPPTz2T	0.06	7 × 10^−3^	[82]
pDPPTz2T-1	0.86	0.014	[82]
pDPPTz2T-2	1.10	0.02	[82]
Boc-TATDPP	3.4 × 10^−6^		[83]
NH-TATDPP	4.2 × 10^−4^		[83]

## Data Availability

Not applicable
